# The Effects of Diabetic Retinopathy Stage and Light Flicker on Inner Retinal Oxygen Extraction Fraction

**DOI:** 10.1167/iovs.16-20048

**Published:** 2018-06

**Authors:** Anthony E. Felder, Justin Wanek, Norman P. Blair, Charlotte E. Joslin, Katherine C. Brewer, Felix Y. Chau, Jennifer I. Lim, Yannek I. Leiderman, Mahnaz Shahidi

**Affiliations:** 1 Department of Ophthalmology & Visual Sciences, University of Illinois at Chicago, Chicago, Illinois, United States; 2 School of Public Health, Division of Epidemiology and Biostatistics, University of Illinois at Chicago, Chicago, Illinois, United States; 3 University of Illinois Cancer Center, Population Health, Behavior, and Outcomes Program, Chicago, Illinois, United States

**Keywords:** diabetic retinopathy, light flicker stimulation, inner retinal oxygen extraction fraction, retina

## Abstract

**Purpose:**

We determined the effects of light flicker and diabetic retinopathy (DR) stage on retinal vascular diameter (D), oxygen saturation (SO_2_), and inner retinal oxygen extraction fraction (OEF).

**Methods:**

Subjects were categorized as nondiabetic control (NC, *n* = 42), diabetic with no clinical DR (NDR; *n* = 32), nonproliferative DR (NPDR; *n* = 42), or proliferative DR (PDR; *n* = 14). Our customized optical imaging system simultaneously measured arterial and venous D (D_A_, D_V_) and SO_2_ (SO_2A_, SO_2V_) before and during light flicker. Inner retinal OEF was derived from SO_2_ values. Light flicker–induced ratios of metrics (D_A_R, D_V_R, SO_2A_R, SO_2V_R, OEFR) were calculated.

**Results:**

Arterial D was larger in NPDR compared to NC (*P* = 0.01) and PDR (*P* = 0.002), whereas D_V_ was similar among groups (*P* ≥ 0.16). Light flicker increased D_A_ and D_V_ (*P* ≤ 0.004), but D_A_R and D_V_R were similar among groups (*P* ≥ 0.09). Arterial SO_2_ was higher in all groups compared to NC (*P* ≤ 0.02) and higher in PDR compared to NDR and NPDR (*P*<0.001). Arterial SO_2_ did not change with light flicker (*P* ≥ 0.1). Venous SO_2_ was higher in NPDR and PDR compared to NC and NDR (*P* ≤ 0.02). Light flicker increased SO_2V_ in NC, NDR, and PDR (*P* ≤ 0.003), and SO_2V_R was lower in NPDR compared to NC and NDR (*P* ≤ 0.05). Inner retinal OEF was lower in NPDR compared to NDR and PDR (*P* ≤ 0.02). Light flicker decreased OEF (*P* ≤ 0.03), but OEFR was greater in NPDR compared to NC and NDR (*P* ≤ 0.03).

**Conclusions:**

The findings of alterations in retinal D, SO_2_, OEF, and their light flicker–induced responses at stages of DR may be useful to elucidate the pathophysiology of DR.

Diabetic retinopathy (DR) is associated with progressive retinal vasculopathy and is a common cause of vision loss.^1,2^ Previous studies have reported that DR significantly affects retinal vessel diameter (D)^3,4^ and oxygen saturation (SO_2_).^5,6^ Furthermore, alterations in retinal arterial and venous D (D_A_, D_V_) have been related to the incidence^7,8^ and progression^3^ of DR.

Light flicker stimulation is an established technique to assess the functional capacity of the retina to respond to a physiological challenge. In healthy human subjects, light flicker has been shown to stimulate retinal neural activity,^9^ augment D,^10,11^ alter SO_2,_^12^ and decrease inner retinal oxygen extraction fraction (OEF).^13^ Inner retinal OEF is defined as the ratio of inner retinal oxygen metabolism (MO_2_) to oxygen delivery (DO_2_). Thus, the light flicker–induced OEF response provides information about the ability of the retinal vasculature to address changes in inner retinal oxygen metabolism.^13^ In DR, reductions in light flicker–induced responses of D^14–16^ and SO_2_^17^ have been demonstrated compared to healthy subjects. However, to our knowledge, neither OEF nor its flicker-induced response has been reported previously in DR. Furthermore, light flicker–induced responses of D, SO_2_, and OEF typically are not assessed simultaneously, potentially affecting results due to intertest variability. Simultaneous assessment of retinal D, SO_2_, and OEF and their flicker-induced responses at stages of DR may be useful to elucidate the pathophysiology of DR. In the current study, we tested the hypothesis that D, SO_2_, OEF, and their light flicker–induced responses are altered at stages of DR.

## Methods

### Subjects

The study was approved by an Institutional Review Board at the University of Illinois at Chicago. Before enrollment, the research study was explained to the subjects and informed consents were obtained according to the tenets of the Declaration of Helsinki. A total of 130 subjects participated in the study. Subjects' eyes were classified by clinical examination as nondiabetic control (NC; *n* = 42), diabetic without clinical retinopathy (NDR; *n* = 32), nonproliferative diabetic retinopathy (NPDR; *n* = 42), or proliferative diabetic retinopathy (PDR; *n* = 14). All PDR subjects had received panretinal photocoagulation (PRP) treatment. Exclusion criteria included history of stroke or myocardial infarction (within 3 months before imaging), active angina, sickle cell disease, glaucoma, age-related macular degeneration, retinal vascular occlusion, refractive error >6 diopters, or intraocular surgery or cataract surgery performed within 9 months of imaging.

Before imaging, subjects' pupils were dilated using 1% tropicamide and 2.5% phenylephrine. Subjects were seated in front of a modified slit-lamp biomicroscope with their heads resting on a chin and forehead support. During imaging, a light emitting diode was presented to the fellow eye as a fixation target. Subjects were continuously light adapted during imaging due to the instrument's retinal illumination light. Retinal imaging was performed before and during light flicker stimulation. One eye per subject was selected based on the exclusion criteria. If both eyes qualified, the eye with better image data was selected.

### Instrumentation

**Figure 1:**
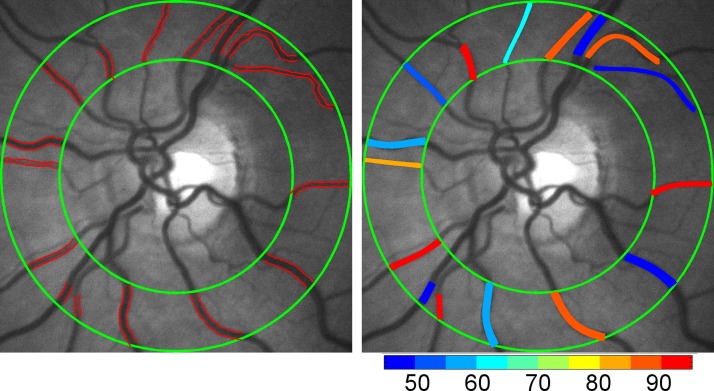
Example of a retinal reflectance image acquired using light at 570 nm wavelength in a representative nondiabetic control subject. Retinal vessels were segmented and a mean diameter was measured for each vessel (red lines) within the circumpapillary region of interest enclosed by two concentric green circles (Left). A mean retinal vascular oxygen saturation was calculated for each vessel using reflectance images acquired with light at 570 and 606 nm wavelengths. Oxygen saturation is overlaid on the retinal image in pseudocolor for illustrative purposes (Right). Color bar: oxygen saturation in percent.

Our previously described optical imaging system was used to simultaneously quantify retinal vascular D and SO_2_ before and during light flicker stimulation.^13^ Briefly, a rapid switching filter wheel was fitted with three bandpass filters and inserted into the illumination path of the slit-lamp biomicroscope. Retinal reflectance images were acquired at 606 and 570 nm wavelengths within 3 seconds before and during light flicker stimulation. Light flicker stimulation was provided at 10 Hz for 60 seconds using light at 530 nm. Images from 606 and 570 nm wavelengths were registered using an automated algorithm and averaged to generate a single mean image at each wavelength. Retinal vessels within a circumpapillary region of interest were segmented (Fig. 1) and vessel centerlines were generated. Vessel D was measured along the vessel centerlines and vessel SO_2_ (Fig. 1) was calculated from optical density ratio measurements. Measurements of D and SO_2_ from each vessel within the circumpapillary region of interest were averaged to yield a mean D_A_ and D_V_, and arterial and venous SO_2_ (SO_2A_, SO_2V_). These mean values were determined for each subject before and during light flicker stimulation.

Inner retinal OEF quantifies the ratio of MO_2_ to DO_2_. With Fick's equation,^18^ MO_2_ and DO_2_ can be expanded using retinal blood flow (BF) and oxygen content. Since the solubility of oxygen in blood is minimal,^19^ SO_2_ is used to estimate oxygen content. Furthermore, since BF is a determinant of MO_2_ and DO_2_, the ratio defined by OEF is independent of BF. Thus, OEF was calculated as [(SO_2A_ − SO_2V_)/SO_2A_]^13^ and used to provide information on the ratio of MO_2_ to DO_2_ without providing absolute measurement of either terms. Inner retinal OEF was calculated before and during light flicker stimulation. Light flicker–induced metric ratios (D_A_R, D_V_R, SO_2A_R, SO_2V_R, and OEFR) were calculated by dividing the value of the metric during light flicker by the value before light flicker.

### Data Analysis

The distributions of metrics D_A_, D_V_, SO_2A_, SO_2V_, and OEF were evaluated to assess data normalcy and identify outliers.^20^ Regression diagnostics including Cook's distance were performed to assess the linear relationship between DR stage and each metric to identify data points that were outliers, had leverage, or were influential. Three outliers were identified, which were removed from further analyses. Subsequent testing for each metric indicated normalcy. The effect of light flicker on measurements of metrics (D_A_, D_V_, SO_2A_, SO_2V_, and OEF) within each DR stage group (an intragroup comparison) was performed by paired *t*-test using metric values before and during light flicker. Descriptive statistics were compared for demographic variables using the χ^2^ test and *t*-tests. The independent effects of DR stage group (an intergroup comparison) on baseline measurements of metrics (D_A_, D_V_, SO_2A_, SO_2V_, OEF) and metric ratios (D_A_R, D_V_R, SO_2A_R, SO_2V_R, and OEFR) were assessed using linear regression analysis. Multivariable linear regression^21^ models were constructed using a priori–selected covariates (age, race, sex, eye examined) from univariate models to compute the parameter estimates and 95% confidence intervals. All statistical tests were 2-sided and significance was set to *P* ≤ 0.05. All statistical analyses were performed using SAS version 9.4 (SAS Institute, Inc., Cary, NC, USA).

## Results

**Figure 2:**
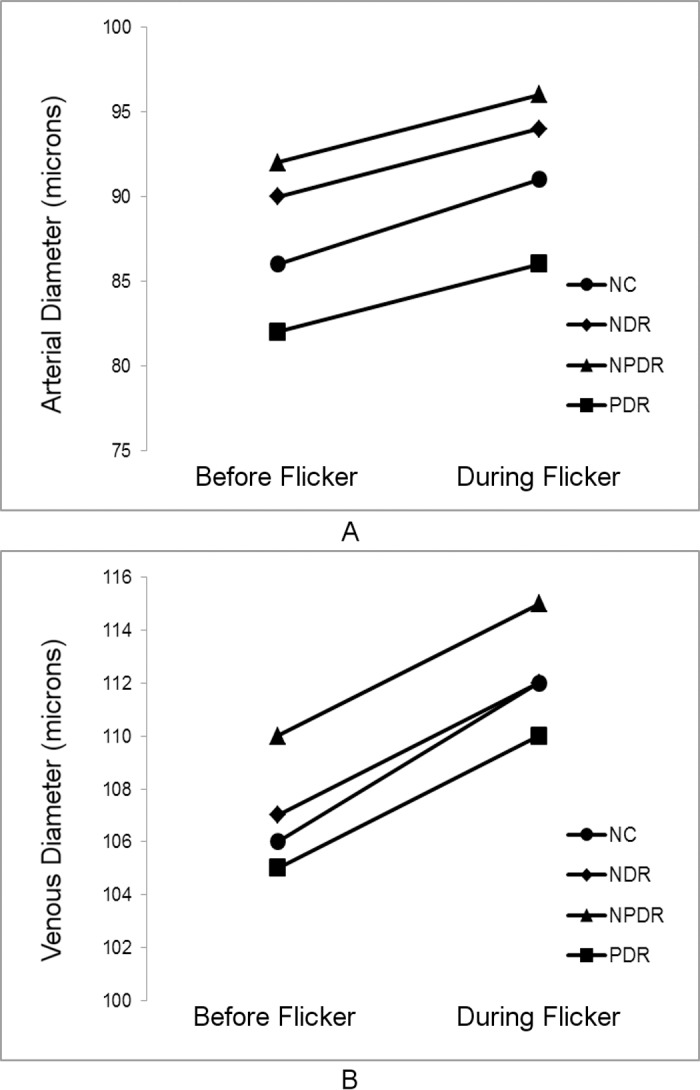
Mean retinal arterial (A) and venous (B) diameter measurements before and during light flicker in each DR stage group.

**Figure 3:**
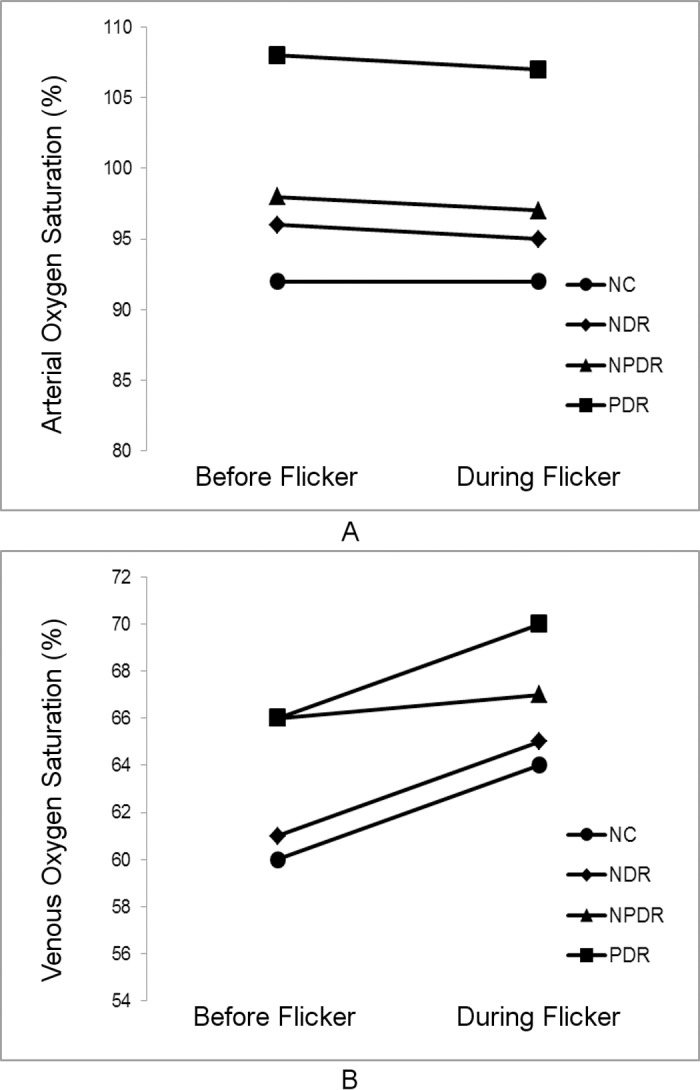
Mean retinal arterial (A) and venous (B) oxygen saturation measurements before and during light flicker in each DR stage group.

**Figure 4:**
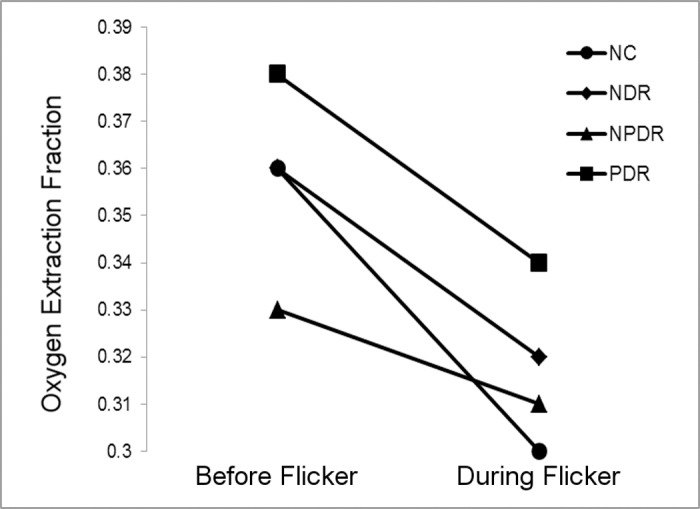
Mean inner retinal oxygen extraction fraction measurements before and during light flicker in each DR stage group.

Demographic data for subjects are presented in Table 1. The distribution of races differed significantly among DR stage groups (*P* < 0.001). Mean ages of NC subjects (59 ± 13 years; mean ± SD), NDR subjects (56 ± 12 years), NPDR subjects (56 ± 10 years), and PDR subjects (51 ± 11 years) were not significantly different (*P* = 0.2). Unadjusted means of metrics (D_A_, D_V_, SO_2A_, SO_2V_, and OEF) before and during light flicker stimulation as well as metric ratios (D_A_R, D_V_R, SO_2A_R, SO_2V_R, and OEFR) stratified by DR stage group are presented in Table 2. For each DR stage group, means were statistically adjusted for covariates (age, race, sex, eye examined). Differences in estimated means of metrics (D_A_, D_V_, SO_2A_, SO_2V_, and OEF) before light flicker between DR stage groups are provided in Table 3. Similarly, differences in estimated means of metric ratios (D_A_R, D_V_R, SO_2A_R, SO_2V_R, and OEFR) between DR stage groups are presented in Table 4. Unadjusted mean metrics (D, SO_2_, and OEF) before and during light flicker stimulation in each DR stage group are shown in Figures 2 to 4, respectively.

**Table 1:**
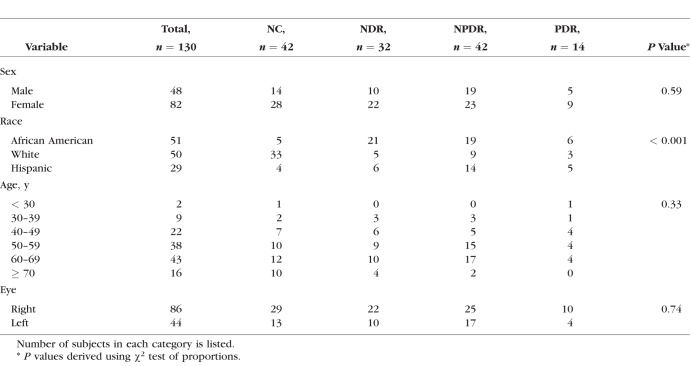
Characteristics of Subjects Stratified by DR Stage Group

**Table 2:**
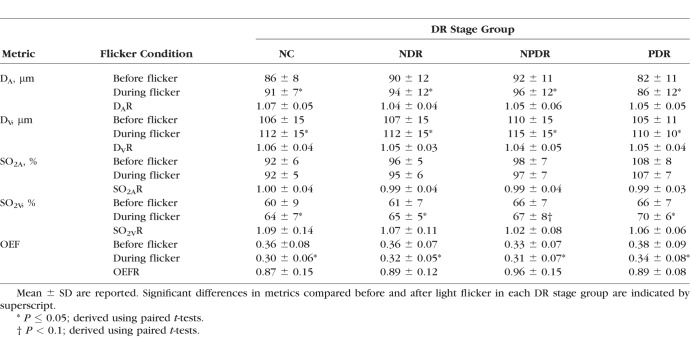
Unadjusted D_A_, D_V_, SO_2A_, SO_2V_, and OEF Before and During Light Flicker Stimulation and Their Light Flicker–Induced Ratios (D_A_R, D_V_R, SO_2A_R, SO_2V_R, and OEFR) Stratified by DR Stage Group

**Table 3:**
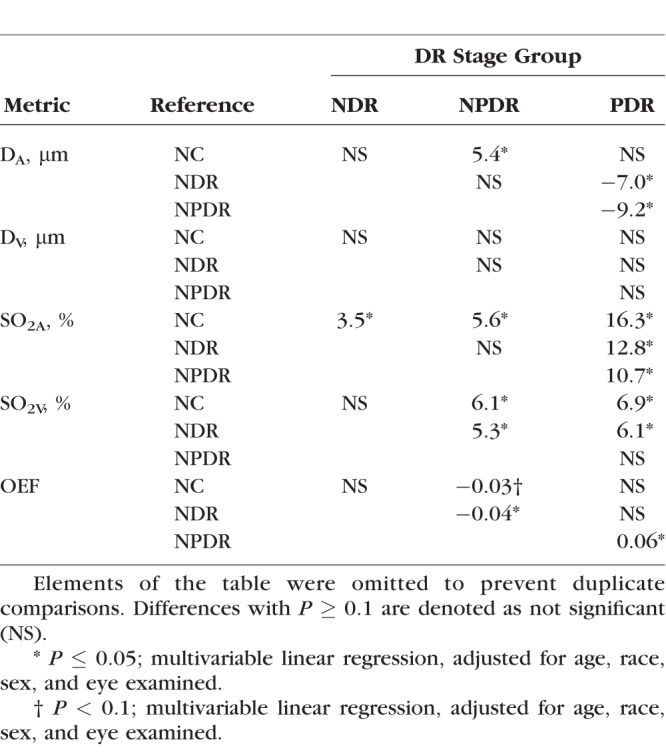
Differences in Estimated Means of D_A_, D_V_, SO_2A_, SO_2V_ and OEF Before Light Flicker Between Each DR Stage Group and a Reference Group

**Table 4:**
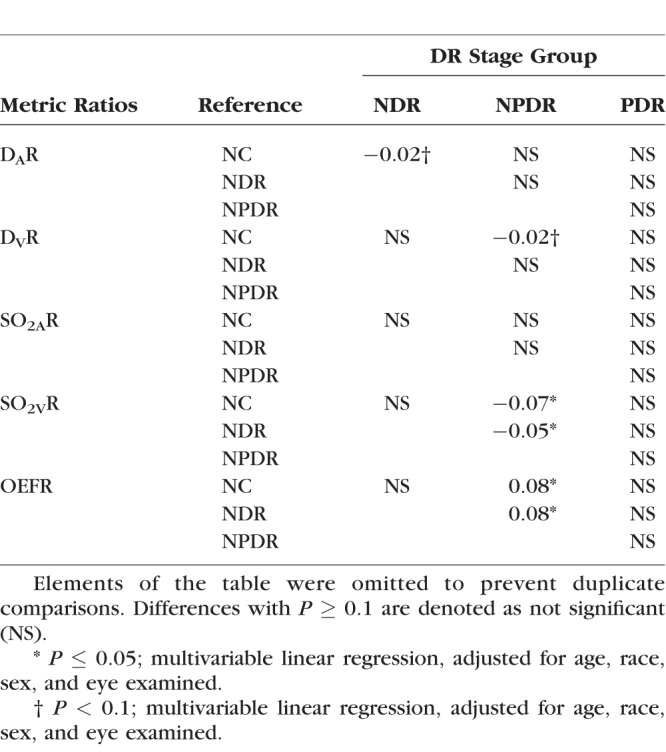
Differences in Estimated Means of D_A_R, D_V_R, SO_2A_R, SO_2V_R, and OEFR Between Each DR Stage Groups and a Reference Group

### Effect of DR Stage: Intergroup Comparison

Differences in estimated means of D_A_, D_V_, SO_2A_, SO_2V_, and OEF before light flicker between DR stage groups are provided in Table 3. Arterial D was significantly larger in NPDR compared to NC and PDR subjects (*P* ≤ 0.01). Arterial D also was larger in NDR compared to PDR subjects (*P* = 0.02). There was no significant difference in D_V_ between DR stage groups (*P* ≥ 0.16). Arterial SO_2_ was higher in NDR, NPDR, and PDR compared to NC subjects (*P* ≤ 0.02). Additionally, SO_2A_ was higher in PDR compared to NDR and NPDR subjects (*P* < 0.001). Venous SO_2_ was higher in NPDR and PDR compared to NC (*P* ≤ 0.01) and NDR subjects (*P* ≤ 0.02). In NPDR subjects, OEF was significantly lower compared to NDR and PDR subjects (*P* ≤ 0.02) and tended to be lower than NC subjects (*P* = 0.07).

### Effect of Light Flicker Stimulation: Intragroup Comparison

Unadjusted means of D_A_, D_V_, SO_2A_, SO_2V_, and OEF before and during light flicker as well as their metric ratios are shown in Table 2. Arterial D and D_V_ significantly increased during light flicker in all DR stage groups (*P* ≤ 0.004; Fig. 2). Arterial SO_2_ did not significantly change during light flicker in all DR stage groups (*P* ≥ 0.1; Fig. 3). Venous SO_2_ significantly increased during light flicker in NC, NDR, and PDR subjects (*P* ≤ 0.003; Fig. 3). The increase in SO_2V_ during light flicker in NPDR subjects approached statistical significance (*P* = 0.07). Since SO_2A_ did not change and SO_2V_ increased with light flicker, OEF significantly decreased during light flicker in all DR stage groups (*P* ≤ 0.03; Fig. 4).

### Effect of Light Flicker Stimulation: Intergroup Comparison

Differences in estimated means of D_A_R, D_V_R, SO_2A_R, SO_2V_R, and OEFR between DR stage groups are provided in Table 4. There was a trend of diminished D_A_R in NDR compared to NC subjects (*P* = 0.09) and diminished D_V_R in NPDR compared to NC subjects (*P* = 0.09). The light flicker–induced ratio of SO_2A_ was similar between DR stage groups (*P* ≥ 0.18). The light flicker–induced ratio of SO_2V_ was lower in NPDR subjects compared to NC and NDR subjects (*P* ≤ 0.05). The light flicker–induced ratio of OEF was higher in NPDR compared to NC and NDR subjects (*P* ≤ 0.03), corresponding to a diminished light flicker–induced OEF response in NPDR.

## Discussion

Using our previously developed optical imaging system, simultaneous measurements of retinal D, SO_2_, and OEF were obtained before and during light flicker in nondiabetic control and diabetic subjects at stages of DR. Metrics before light flicker stimulation and their light flicker–induced responses were compared after adjusting for age, race, sex, and eye examined. The results confirmed our hypothesis that D, SO_2_, and OEF and their light flicker–induced responses are altered at stages of DR. Arterial D was increased in NPDR and decreased in PDR and the light flicker–induced vasodilatory responses tended to be decreased in NDR and NPDR. Arterial SO_2_ and SO_2V_ were increased in NDR, NPDR, and PDR and the light flicker–induced increase in SO_2V_ was diminished in NPDR. Correspondingly, in NPDR subjects, OEF was decreased and the light flicker–induced decrease in OEF was diminished.

### Effect of DR Stage: Intergroup Comparison

In the current study, D_A_ was significantly larger in NPDR compared to NC and PDR subjects. This finding is consistent with previous studies that found that retinal arterial dilation is related to the incidence of DR.^7,8,22^ However, D_V_ was not significantly different among DR stage groups in the current study, in contrast to the findings of Klein et al.^3^ and Kifley et al.,^22^ who reported that dilation of retinal veins was related to DR progression. The discrepancy between these results is likely due to the smaller sample size in the current study, study design, and differences in covariate corrections. In the current study, SO_2A_ was higher in all DR stage groups compared to NC subjects. Additionally, SO_2A_ was higher in PDR compared to NDR and NPDR subjects. Venous SO_2_ was higher in NPDR and PDR compared to NC and NDR subjects. These findings are in agreement with previous studies that found increased SO_2A_ and SO_2V_ in DR^6,23^ and increasing SO_2V_ with DR progression.^5,23,24^

In NPDR subjects, OEF was significantly lower compared to NDR and PDR, and tended to be lower than NC subjects. Since OEF is defined as the ratio of MO_2_ to DO_2_,^13^ these results indicated differences in MO_2_ and DO_2_. Inner retinal oxygen DO_2_ is determined by arterial blood oxygen content and BF, which is, in turn, related to D. Although DO_2_ was not directly measured in the current study, DO_2_ was likely increased in NPDR since D_A_ was larger, consistent with previous reports of increased BF in NPDR subjects.^25,26^ On the other hand, MO_2_ may have been reduced during NPDR. Retinal hypoxia is implicated in DR,^27,28^ which depending on severity, may cause a reduction in MO_2_ as shown under severe hypoxia in rats.^29^ However, a decrease in MO_2_ is only speculative, since direct measurement of MO_2_ has not been reported previously in DR subjects, to our knowledge. Therefore, it seems likely that the observed reduction of OEF in NPDR was primarily due to an increase in DO_2_. In PDR subjects, OEF was not significantly different from that of NC subjects. Since all PDR subjects had received PRP treatment, inner retinal oxygenation was presumably improved^30^ due to a loss of oxygen-consuming outer retinal tissue and the resultant increased oxygen flux from the choroidal circulation. Thus, as a result of increased oxygen delivery from the choroid, DO_2_ is expected to decrease as the retinal circulation autoregulates. Furthermore, due to the increase in oxygen availability from the choroid and presumably less retinal tissue, inner retinal MO_2_ is also decreased. Therefore, the finding of similar OEF between PDR and NC subjects is consistent with decreases to MO_2_ and DO_2_.

### Effect of Light Flicker Stimulation: Intragroup Comparison

Increased D_A_ and D_V_ in response to light flicker is in agreement with previous studies that demonstrated retinal vasodilation in NC,^12,13^ NDR,^14,27^ NPDR,^14,17^ and PDR subjects.^14^ Arterial SO_2_ did not respond to light flicker in all DR stage groups, whereas SO_2V_ significantly increased in NC, NDR, and PDR subjects, and tended to increase in NPDR subjects. These results are in agreement with previous studies that found light flicker did not change SO_2A_ and increased SO_2V_ in NC^12,13^ and NPDR subjects.^17^ In the current study, OEF decreased with light flicker in all DR stage groups, consistent with our previous study.^13^ A decrease in OEF indicates that the light flicker–induced augmentation in DO_2_ was greater than the respective light flicker–induced change in MO_2_.^13^ This finding is in agreement with a recent study by Palkovits et al.^31^ which demonstrated that light flicker–induced increases in BF (55%) exceeded that of oxygen extraction (35%) in NC subjects.

### Effect of Light Flicker Stimulation: Intergroup Comparison

The light flicker–induced ratio of D_A_ tended to be lower in NDR compared to NC subjects, in agreement with previous studies using the Dynamic Vessel Analyzer.^27,28,32^ The light flicker–induced ratio of D_V_ tended to be lower in NPDR compared to NC subjects. The vasodilatory findings in the current study are not consistent with previous studies that reported a progressive reduction in D_A_R^33^ and D_V_R^14^ with DR stage. Differences in findings may be attributed to the smaller sample size in the current study and differences in covariate corrections.

The light flicker–induced ratio of SO_2A_ was not different among DR stage groups, while SO_2V_R was lower in NPDR compared to NC and NDR subjects, in agreement with a previous study.^17^ One possible explanation for this observation is based on reduced availability of oxygen to the retinal tissue in NPDR. In the nondisease state, the retinal tissue receives sufficient oxygen and, thus, during light flicker–induced augmentations of DO_2_, abundant amounts of oxygen are delivered that exceed the change in metabolic demand of the tissue.^31^ This results in an increase of SO_2V_ with light flicker and an SO_2V_R greater than unity. In contrast, tissue that receives insufficient oxygen will extract more when it is made available during the light flicker–induced augmentation of DO_2_. This causes a diminished increase in SO_2V_ and a decreased SO_2V_R compared to normal tissue. Indeed, the vascular pathologies of NPDR, such as capillary shunting^34^ and nonperfusion on fluorescein angiography,^35,36^ indicate decreased DO_2_. In contrast, SO_2V_R was not significantly different between NC, NDR, and PDR subjects. Subjects with NDR likely had normal retinal oxygenation, supported in part by the lack of visible vascular pathologies. All PDR subjects had received PRP treatment which likely improved their inner retinal oxygenation such that abundant amounts of oxygen were available during light flicker, resulting in an SO_2V_R similar to that of NC subjects.

The light flicker–induced ratio of OEF quantifies the ratio of light flicker–induced responses in MO_2_ to DO_2_ without directly quantifying either response. The light flicker–induced ratio of OEF was not significantly different between NC and NDR subjects, despite an observed trend of impaired vasodilatory response, suggesting a diminished light flicker–induced response in DO_2_. Together, these results suggested a diminished light flicker–induced response in MO_2_ before the development of NPDR. The light flicker–induced ratio of OEF was higher in NPDR compared to NC and NDR, which is likely due to a diminished light flicker–induced response in DO_2_, consistent with the observed trend of impaired vasodilatory response. However, a change in the light flicker–induced response of MO_2_ cannot be excluded_._ Interestingly, OEFR and the vasodilatory responses to light flicker were not significantly different between NC and PDR subjects. This result suggests that PRP treatment may promote the restoration of light flicker–induced responses in DO_2_ and MO_2._

There were several limitations in the current study. First, OEF quantifies the ratio of MO_2_ to DO_2_ and cannot directly quantify either quantity due to a lack of BF measurements. Future studies that simultaneously measure MO_2_ and DO_2_ in stages of DR are needed to elucidate the underlying reason for a reduced OEF and its flicker-induced response in DR. Second, since data were acquired by optical imaging techniques, image quality may have affected measurements. However, the system was validated previously and shown to be capable of detecting light flicker–induced changes.^13^ Third, a fixed calibration factor was used to calculate vessel diameters^13^ and, thus, did not account for variations in refractive error among subjects. However, subjects with high refractive error, greater than 6 diopters, were excluded from the study. Furthermore, the use of a constant calibration did not affect diameter measurements compared within subjects or flicker-induced diameter ratios compared between subjects. Fourth, there were variations in the clinical history and status of DR subjects. Future studies with a larger sample size that can account for clinical confounding factors are needed to substantiate the current findings and reveal differences not discernable with this sample size.

In conclusion, vessel diameters were larger at stages of DR and the flicker-induced changes tended to be decreased. Oxygen saturation of vessels increased at stages of DR and the flicker-induced changes in SO_2V_ were different in NPDR. Correspondingly, OEF and OEFR were decreased in NPDR, suggesting impairment of the MO_2_ and DO_2_ and their responses to light flicker in DR. These findings of alterations in D, SO_2_, and OEF and their light flicker–induced responses may help to elucidate the pathophysiology of DR.
